# How to Build a Simulation Center for Surgery

**DOI:** 10.1177/15569845231201618

**Published:** 2023-09-29

**Authors:** Shokoufeh Cheheili Sobbi, Peyman Sardari Nia

**Affiliations:** 1 Department of Cardiothoracic Surgery, Heart and Vascular Centre, Maastricht University Medical Centre, The Netherlands

## Introduction

The evolution of work patterns, the paramount importance of patient safety, the increase in the complexity of surgical procedures, and rapid introduction of new surgical techniques has forced the development of simulation platforms. Substantiated by evidence in previous research, simulation-based training has emerged as a potent avenue for acquiring surgical expertise.^1–5^ In lieu of traditional trial-and-error learning, simulation-based training offers a structured pathway to mastering surgical skills. Simulation training should be an integral component of surgical education to achieve both fundamental and advanced surgical competencies.

Successful adoption of a new procedure will be enhanced by a training concept rather than a single course. The first step toward creating an effective simulation center is careful organization. Clear goals and well-defined objectives are fundamental for initiating a successful program. Establishing a dedicated team of experts, including surgeons, educators, and technology specialists, ensures that the center’s activities are aligned with educational objectives while leveraging cutting-edge tools and resources.

In this article, we present a comprehensive guide to building a successful simulation center. Drawing on evidence-based educational principles and our own experience, this article outlines key components, strategies, and considerations essential for creating a state-of-the-art simulation center that fosters excellence in surgical practice.

## Curriculum Building

A critical initial step involves assessing building a curriculum based on the educational needs of the target audience, whether medical students, residents, or practicing surgeons. Clear and achievable goals must be established to guide the program’s development and ensure alignment with educational objectives. The latest educational science should be applied, and the curriculum should be constructed with clear objectives. Amalgamating awareness, skill acquisition with necessary instruments, and theoretical knowledge are the basic learning objectives. This holistic approach equips participants with a nuanced skill set, encompassing both technical skills and cognitive proficiency.

Our preferred curriculum design is based on backwards chaining.^6,7^ Backward chaining is a strategy that involves starting from a desired goal and working backward to determine the sequence of steps or actions needed to achieve that goal. We must ask ourselves why we invest energy, time, and resources into any educational program. What objective do we hope to achieve? Once the objectives are defined, we should develop an assessment tool to determine whether the objectives are met. Only after defining the objectives and crafting the assessment tools should we decide on the course format in terms of its duration and whether it should be theoretical, hands on, or both. After deciding the format, we develop the content and, finally, select the faculty.

In addition to curriculum for surgical skills training, we should consider integrating the skills part within a comprehensive course about procedural steps, tips, tricks, organizational matters, team building, and pitfalls. Hence, simulation-based training should serve as a tool to facilitate procedural learning. On its own, any simulator, whether high or low fidelity, offers limited educational value and impact.

## Use of Validated Simulators

In recent years, a range of simulators and training models have emerged.^8^ These tools could offer a structured environment for skill development, ensuring objectivity and replicability in training. An ideal simulation should be realistic and mimic the setup for a procedure. The use of a validated simulator, with evidence regarding its usability and efficacy, is more effective on surgical skills. The concept of fidelity within simulation is intricate and encompasses various dimensions, primarily tethered to the level of realism achieved through equipment, scenarios, settings, and precision.

Any educational platform or model incorporated within a course must undergo validation or draw from experiences and data to determine the educational significance of the simulators.^9^ The validation process for simulation platforms should be considered at 3 levels in terms of its realism, utility and ability to simulate desired tasks, and its educational value and impact on clinical practice. Furthermore, the platform should ideally have a feedback system and provide real-time feedback, which is a critical component of any training program centered around simulation.

**Fig. 1.**
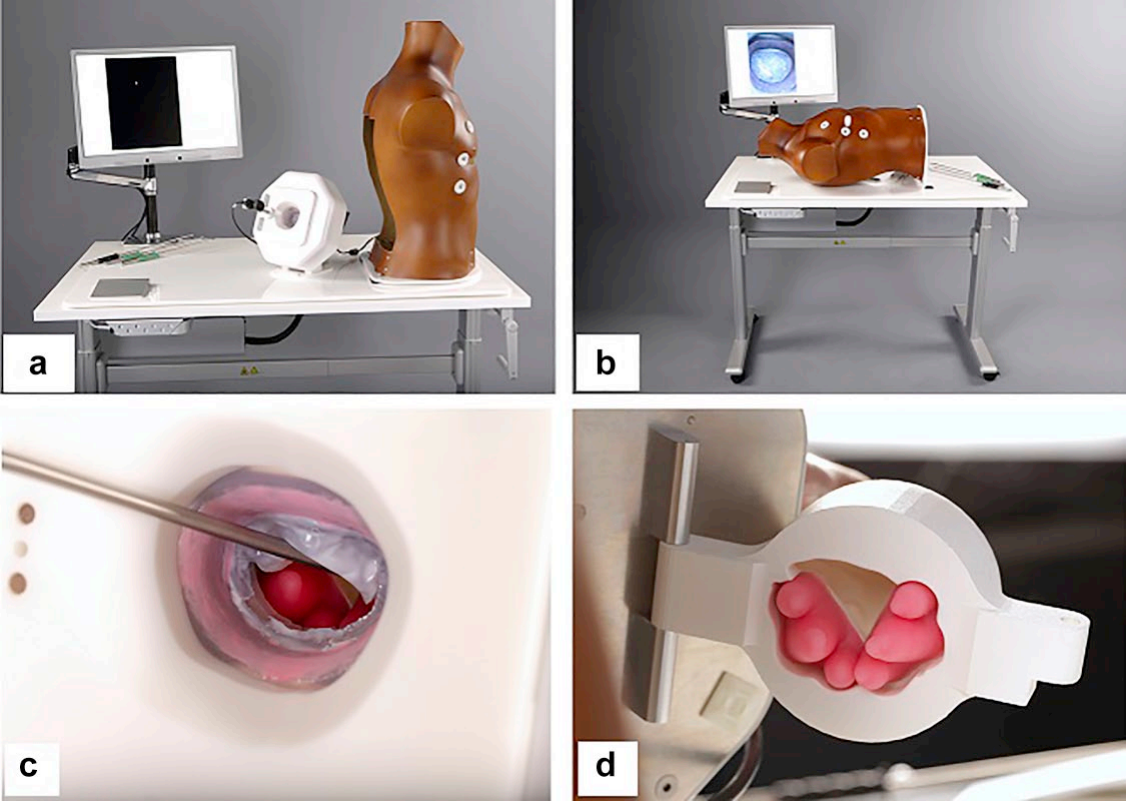
Minimally Invasive Mitral Valve Surgery (MIMVS) simulator. (a) (b) View of the assembled simulator. (c) (d) Magnetic, silicone papillary muscles mounted in the 3-dimensional–printed ventricle. Reprinted from *J Thorac Cardiovasc Surg*, Volume 157, Nia PS et al., Development of a high-fidelity minimally invasive mitral valve surgery simulator, pgs 1567–1574, ©2019, with permission from Elsevier.^10^

**Fig. 2.**
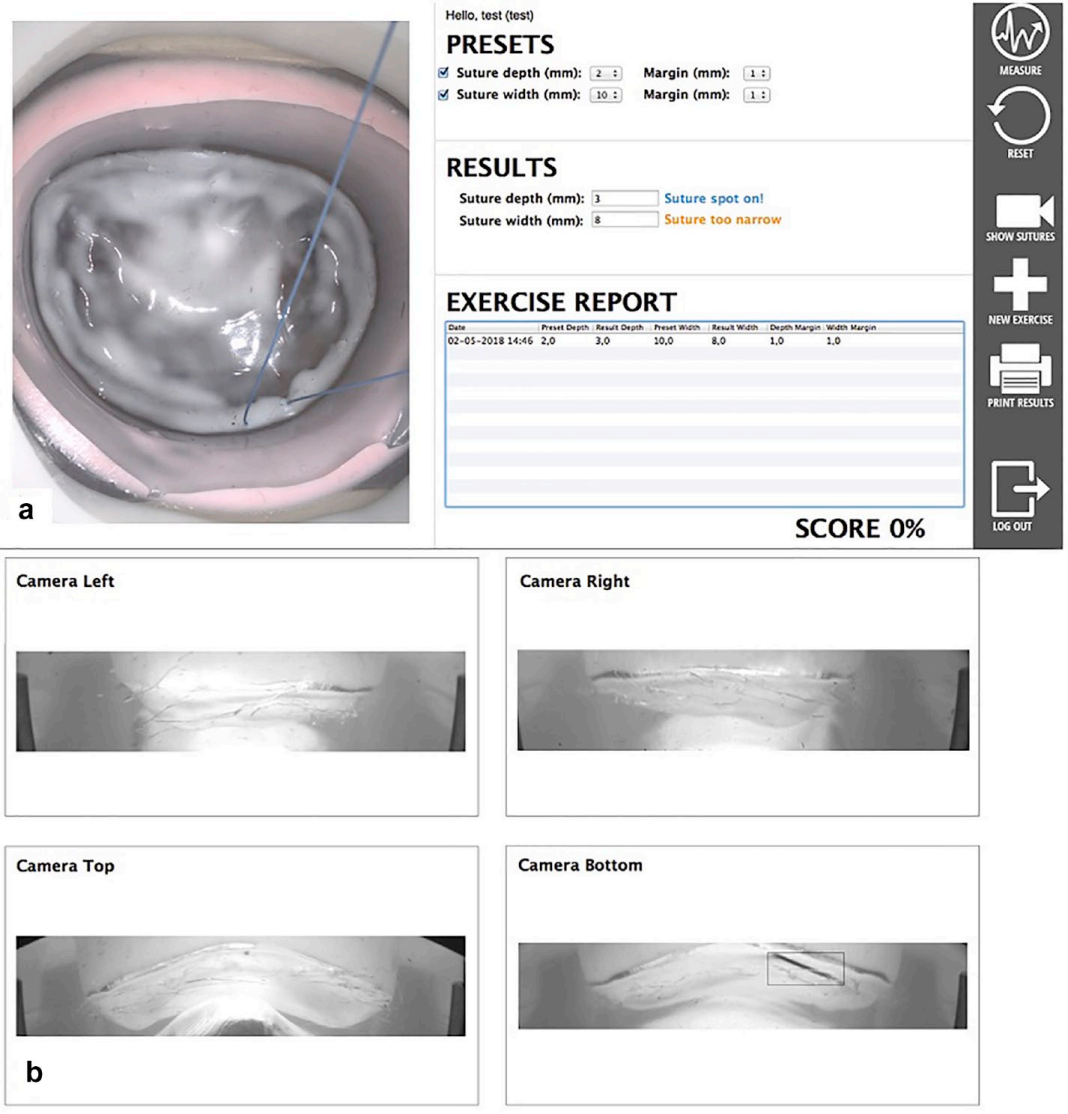
User interfaces of the feedback system. (a) Graphical user interface that provides on-screen feedback, the endoscopic view, and allows the user to define the margin of error. (b) Internal camera view of the feedback system from which the width and depth are measured. Reprinted from *J Thorac Cardiovasc Surg*, Volume 157, Nia PS et al., Development of a high-fidelity minimally invasive mitral valve surgery simulator, pgs 1567–1574, ©2019, with permission from Elsevier.^10^

**Fig. 3.**
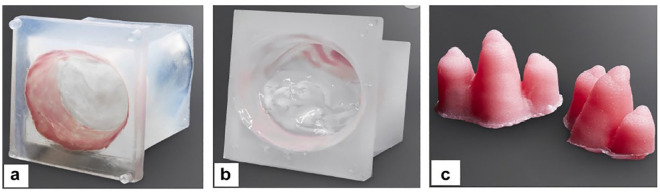
Three-dimensional silicone-cast mitral valve model. (a) Normal mitral valve. (b) Pathological mitral valve. (c) Papillary muscles.

For our simulation center, we have designed an advanced high-fidelity simulator tailored specifically for endoscopic mitral valve surgery, known as the Minimally Invasive Mitral Valve Simulator (MIMVS; Fig. 1, Supplemental Video 1).^10^ This innovative simulator offers real-time feedback functionality, particularly pertaining to the accuracy of suture placements in terms of both width and depth (Fig. 2).^10^ We have also developed a disposable 3-dimensional (3D)–printed pathological silicone replica that can be mounted into the simulator so that one can also be trained in any repair technique on any pathology of the mitral valve (Fig. 3).^6,10^ This replica can be seamlessly integrated into the simulator, enabling comprehensive training across various repair techniques applicable to diverse mitral valve pathologies. The MIMVS simulator facilitates skill enhancement, aids in the strategic planning of complex cases, and serves as a valuable tool for initiating new endoscopic mitral valve surgery programs.

The simulator measurements of our MIMVS simulator have undergone meticulous calibration and validation procedures. Our MIMVS simulator is likely the only simulator in cardiac surgery that is validated at 3 requisite levels in terms of its realism, utility, and ability to simulate the desired tasks and its educational value for simulation-based training.^6,10^ These efforts have substantiated the educational efficacy of our MIMVS simulator, affirming its capability to teach participants the knowledge and skills for endoscopic skills needed for MIMVS. This validation has been further bolstered through comprehensive evaluation by independent surgical experts.^6^ The unanimous consensus among these evaluators is that the high-fidelity MIMVS simulator impeccably replicates the visual and tactile realism inherent to actual endoscopic mitral valve surgery.

During a simulation training, the presence of a surgical coach assumes a pivotal role by offering invaluable guidance, precise corrections, constructive formative feedback, and adept identification of specific areas of vulnerability necessitating additional practice or targeted improvement. In the context of our simulation program, while the MIMVS simulator lacks the capability to provide feedback on the correctness of suture positioning, participants benefit from practicing under the watchful eye of an experienced supervisor. This seasoned professional serves as a vital point of reference, enriching the training process with their expertise and facilitating a comprehensive learning experience.

**Fig. 4.**
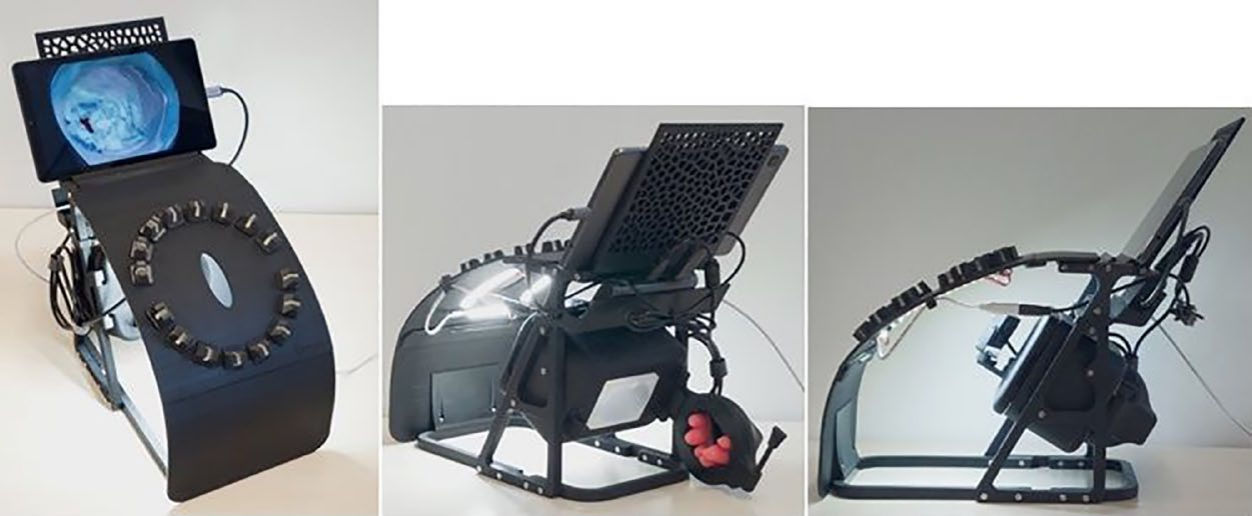
Different views of the telesimulation platform.

To enhance accessibility and mitigate the logistical challenges associated with travel and its associated expenses, we have innovated and validated a novel telesimulation platform for our training program (Fig. 4, Supplemental Video 2). Through this technological advancement, we empower surgeons with the opportunity to engage in our training program without the constraints of geographical distance. Moreover, participants are equipped with the simulator device, affording them the flexibility to engage in practice sessions during a period for continuous practice outside the course.

## Standardization

**Fig. 5.**
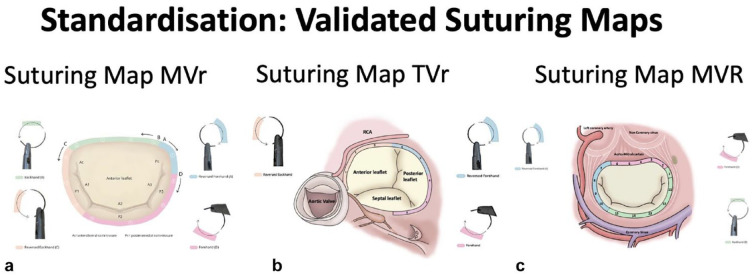
Suturing maps for (a) endoscopic MVr, (b) TVr, and (c) MVR.^11–13^ MVr, mitral valve repair; MVR, mitral valve replacement; TVr, tricuspid valve repair.

Acquiring novel skills should adhere to a consistent and replicable approach. With this objective in mind, we have developed suturing maps tailored to our MIMVS simulator, encompassing techniques for mitral valve repair, mitral valve replacement, and tricuspid valve repair.^11–13^ In the absence of a standardized suturing method for the endoscopic approach, an inexperienced surgeon would need to acquire knowledge through patient-based experimentation, where they would iteratively determine the most efficient suturing sequence, the optimal needle position within the needle holder, and the proper tissue manipulation techniques. Our MIMVS simulator allows us to experiment with different angles and positions of the needle in the needle holder, in combination with different suture sequences, with and without the use of a grasper. In addition, our suturing maps serve as an invaluable aid, facilitating suture placement with minimal tissue manipulation, thereby optimizing surgical efficiency and operative precision. Once the most effective suturing sequences and technique were identified, allowing surgeons to place all sutures with maximal visual exposure and minimal manipulation of the tissue with a grasper, we produced suturing maps (Fig. 5). These suturing maps were then used in the operating room to evaluate intraoperative applicability. Furthermore, the illustrated suturing maps were used in our endoscopic mitral valve courses and helped to standardize the teaching of surgeons learning MIMVS techniques. All participants showed improvement in the correct placement of the annuloplasty sutures and increased their handling speed.

## Stepwise Approach to Teaching Skills

Every surgical procedure involves numerous steps and varying levels of intricacy. Consequently, each simulation training requires a systematic step-by-step approach. For our simulation program on endoscopic mitral valve repair, we defined the key operational steps, which include minimal access, peripheral cannulation, conditioning preparation, heart exposure, mitral exposure, mitral valve repair, and closure.^14^ Our simulation program provides the opportunity to train critical skills for performing these key steps. Our course begins with an orientation to the program and MIMVS simulator, followed by familiarization with equipment such as the use of long-shafted instruments, automatic suturing device, and knot pusher. This is followed by the mastery of fundamental skills, for example, placing sutures at the mitral valve annulus and papillary muscles. Our training is divided into modules, and each module focuses on a specific aspect of surgical skills. Subsequently, the training progresses to encompass more complex skills and competencies, culminating in mitral valve repair on a 3D-printed replica of a pathologic mitral valve annuloplasty with a ring.^10^

## Formative and Metric-Based Feedback

Simulation training for surgery often involves providing formative and metric-based feedback to trainees to enhance their skills and performance. Formative and metric-based feedback plays a crucial role in this process by offering insightful guidance and measurable assessments.

Formative feedback is focused on providing constructive guidance and support during the learning process. In the context of our simulation training, formative feedback aims to help trainees improve their endoscopic surgical skills by highlighting their strengths and areas for improvement. This feedback is given during and immediately after each simulation session by the surgical coach.^6^

Metric-based feedback involves the use of quantitative measures to evaluate a trainee’s performance. We have derived these metrics from real-time feedback technology integrated in the MIMVS simulator.^6,10^ This real-time feedback system provides objective data about various aspects of the trainee’s performance such as accuracy and speed for placing a suture at the mitral valve annulus. Therefore, participants perform a theoretical pre-assessment at the start of the course, consisting of standardized questions regarding endoscopic mitral valve surgery. Participants also perform a practical pre-assessment on surgical skills that is focused on the speed and the accuracy of placing a suture at the anterior and posterior mitral valve annulus. Suture accuracy is scored by the entrance and exit of the needle in the mitral valve annulus and the width of the suture (Supplemental Video 1). The sutures are measured in width by the validated camera measuring system of the MIMVS simulator with a preset width of 10 mm and a margin of error of 2 mm (Fig. 2). Sutures placed with a width of 10 ± 2 mm are considered adequate. At the end of the course, participants perform the same theoretical and technical assessment to determine knowledge and skill gained. As described by our prior study, our participants showed a significant improvement in theoretical and surgical skills after completing the training.^6^

## Measuring and Evaluating the Teaching Experience

Measuring and evaluating the teaching experience in simulation training is crucial for ensuring the effectiveness of the training program and making continuous improvements. It involves assessing various aspects of the teaching process, instructor performance, trainee engagement, and overall learning outcomes. Assessment plays a crucial role in every educational program. It serves as a mechanism to measure the efficacy of a course via both pre- and post-assessment techniques, ensuring the attainment of course objectives.^6^ In parallel with assessment, course evaluation by participants enables educators to refine the program based on learner needs. In addition, the measurement and documentation of participant experiences facilitate a systematic exploration of the educational process, thereby fostering long-term improvements. In our simulation program, participants engage in hands-on pre- and post-assessment sessions, encompassing identical technical skills. In addition, prior to the course, participants complete a theoretical pre-assessment comprising standardized inquiries related to endoscopic mitral valve surgery. Upon course conclusion, participants undertake identical theoretical and technical assessments to gauge the knowledge and skills acquired.^6^

As highlighted before, successful adoption of a new procedure will be enhanced by a training concept rather than a single course. Therefore, evaluation of the training, improvement, and keeping up to date with new educational strategies and new technologies is very important. Our simulation courses are evaluated through a questionnaire that involves designing a comprehensive set of questions that assess various aspects of the course including content, instructional methods, learning outcomes, and participant satisfaction.^6^ Follow-up of the long-term impact of the simulation training on trainee surgical skills is accomplished through the completion of a questionnaire by the trainees.

In addition, we have published research papers and our findings related to the development, validation, and efficacy of our simulation-based training program for endoscopic mitral valve surgery.^6,8,10–15^ This sharing of knowledge facilitates the utilization of our insights by others, thereby making a meaningful contribution to the wider domain of medical education and enhancing the collective knowledge base.
